# Characterization of Two Monoclonal Antibodies That Recognize Linker Region and Carboxyl Terminal Domain of Coronavirus Nucleocapsid Protein

**DOI:** 10.1371/journal.pone.0163920

**Published:** 2016-09-30

**Authors:** Xin Zhang, Xin Zhao, Hui Dong, Yunnuan Zhu, Hongyan Shi, Jianfei Chen, Da Shi, Li Feng

**Affiliations:** 1 State Key Laboratory of Veterinary Biotechnology, Harbin Veterinary Research Institute of the Chinese Academy of Agricultural Sciences, Harbin, Heilongjiang, China; 2 Molecular Biology, Gembloux Agro-Bio Tech, University of Liège, Liège, Belgium; New York Blood Center, UNITED STATES

## Abstract

The transmissible gastroenteritis virus (TGEV) nucleocapsid (N) protein plays important roles in the replication and translation of viral RNA. The present study provides the first description of two monoclonal antibodies (mAbs) (5E8 and 3D7) directed against the TGEV N protein linker region (LKR) and carboxyl terminal domain (CTD). The mAbs 5E8 and 3D7 reacted with native N protein in western blotting and immunofluorescence assay (IFA). Two linear epitopes, ^189^SVEQAVLAALKKLG^202^ and ^246^VTRFYGARSSSA^257^, located in the LKR and CTD of TGEV N protein, respectively, were identified after truncating the protein and applying a peptide scanning technique. Using mAb 5E8, we observed that the N protein was expressed in the cytoplasm during TGEV replication and that the protein could be immunoprecipitated from TGEV-infected PK-15 cells. The mAb 5E8 can be applied for different approaches to diagnosis of TGEV infection. In addition, the antibodies represent useful tools for investigating the antigenic properties of the N protein.

## Introduction

The four Coronavirus (CoV) genera, *alpha-*, *beta-*, *gamma-*, and *deltacoronavirus* are clustered in the *Coronavirinae* subfamily [1, 2]. CoVs are pleomorphic, enveloped, single-stranded, positive-sense RNA viruses, with genomes ranging from 26.2 to 31.7 kb [3, 4]. The genomes of CoVs encode four structural proteins: spike (S), membrane (M), envelope (E), and nucleocapsid (N).

The N protein has been characterized and functions predominantly in the formation of viral ribonucleoprotein (RNP) [5]. CoV N proteins interact with viral-specific RNAs or RNA intermediates and might play important roles during viral transcription and replication [6, 7]. These proteins might also act as RNA chaperones, involved in template switching and required for efficient transcription [8, 9]. Four distinct domains are present in CoV N proteins: intrinsically disordered regions (IDRs), an amino-terminal domain (NTD), a carboxy-terminal domain (CTD), and the linker region (LKR) [10–13]. The LKR of the N protein might play an essential role in the transformation of the N protein and its interaction with viral RNA [14]. The higher order oligomers of the CoV N protein are mediated by the CTD [15, 16]. However, there are few studies to date on the functions of the LKR and CTD. To further dissect the functions of the N protein LKR and CTD, mAbs to the N protein are needed.

In the present study, we described two mAbs, namely 5E8 against the TGEV N protein LKR and 3D7 against the TGEV N protein CTD. Two linear epitopes ^189^SVEQAVLAALKKLG^202^ and ^246^VTRFYGARSSSA^257^ were subsequently identified. Moreover, using mAb 5E8, the N protein was visualized via immunofluorescence assay (IFA) and immunohistochemistry (IHC). The data reported here indicate that 5E8 and 3D7 will be useful for unraveling the functions of the TGEV N protein.

## Materials and Methods

### Cells and virus

Porcine kidney 15 (PK-15) cells were obtained from American Type Culture Collection (ATCC). PK-15 cells were grown in Dulbecco’s minimum essential medium (DMEM) medium supplemented with 5% fetal calf serum under standard culture conditions (5% CO_2_, 37°C). TGEV infectious strain H (Accession No. FJ755618) was propagated on a PK-15 cell monolayer. Porcine epidemic diarrhea virus (PEDV) strain CV777 (Accession No. AF353511) was maintained in the lab.

### Cell infection

PK-15 cells were infected with TGEV infectious strain H at a multiplicity of infection (MOI) of 0.1. After adsorption for 1 h, the cells were washed and incubated in fresh DMEM.

### Construction of recombinant expression plasmids

Plasmid pGEX-TGEV-N was constructed as previously described [17]. Three partial N genes, corresponding to amino acids (aa) 1–141 (nt 1–423), 142–240 (nt 424–720), and 241–382 (nt 721–1179) of TGEV N protein, were amplified using a panel of primers containing *Bam* HI and *Xho* I enzyme sites, as described in Table 1. The PCR products were subcloned into a prokaryotic expression pGEX-6p-1 vector. The recombinant expression plasmids were designated as pGEX-TGEV-N1 (aa 1–141), pGEX-TGEV-N2 (aa 142–240), and pGEX-TGEV-N3 (aa 241–382).

**Table 1:** Primers for the construction of recombinant expression plasmids.

Name	Sequence (5′-3′)	Enzyme
F-GST-N1	CAGGATCCGCCAACCAGGGACAACGT	*Bam* HI
R-GST-N1	CACTCGAGGAATTTCAAAGCTTTGGAT	*Xho* I
F-GST-N2	CAGGATCCGATGGTAAAGTGCCAGGC	*Bam* HI
R-GST-N2	CACTCGAGAGTTCTCTTCCAGGTGTGTT	*Xho* I
F-GST-N3	CAGGATCCGCAGGTAAAGGTGATGTGA	*Bam* HI
R-GST-N3	CACTCGAGGTTCGTTACCTCATCAATCA	*Xho* I
F-GFP-N	GTGAATTCATGGCCAACCAGGGACAACGT	*Eco* RI
R-GFP-N	GTGGATCCTTAGTTCGTTACCTCATCAATCA	*Bam* HI
F-TGEV-N-565-606	AATTCAGTGTAGAACAAGCTGTTCTTGCCGCACTTAAAAAGTTAGGTG	
R-TGEV-N-565-606	GATCCACCTAACTTTTTAAGTGCGGCAAGAACAGCTTGTTCTACACTG	
F-TGEV-N-736-771	AATTCGTGACAAGATTTTATGGAGCTAGAAGCAGTTCAGCCG	
R-TGEV-N-736-771	GATCCGGCTGAACTGCTTCTAGCTCCATAAAATCTTGTCACG	

The full-length N gene was amplified using the primers F-GFP-N and R-GFP-N (Table 1). The purified PCR products were inserted into the eukaryotic expression vector pEGFP-c2 (Clontech Laboratory Inc., Mountain View, CA, USA). Sequences encoding aa189-202 (nt 565–606) and aa 246–257 of the N protein (nt 736–771) were produced by annealing complimentary primers (Table 1); the sequences were inserted into the eukaryotic expression vector pEGFP-c2. The recombinant plasmids were named pEGFP-TGEV-N, pEGFP-TGEV-N-189-202, and pEGFP-TGEV-N-246-257. pEGFP-TGEV-N, pEGFP-TGEV-N-189-202, pEGFP-TGEV-N-246-257, and pEGFP-c2 were purified and transfected into PK-15 cells grown in 96-well plates using Attractene Transfection reagent (Qiagen, China) according to the manufacturer’s instructions.

### Expression and purification of the recombinant protein

Proteins were expressed in *E*. *coli* BL21 (DE3) cells as previously described [17]. The fusion protein was purified using Glutathione Sepharose 4B (GE Healthcare, UK) according to the manufacturer’s instructions.

### Preparation and characterization of mAbs against N protein

Two mAbs against N protein were prepared as previously described [18]. The IgG subtype analysis of the mAb was performed using the SBA Clonotyping System/horseradish peroxidase (HRP) (Southern Biotechnology Associates, Inc., Birmingham, AL, USA).

### Immunoperoxidase monolayer assay (IPMA)

After transfection with pEGFP-TGEV-N-189-202 or pEGFP-TGEV-N-246-257, PK-15 cells were fixed with paraformaldehyde (4%) for 20 min at 4°C. The cells were blocked with 5% skim milk and incubated with the primary antibody (mAb 5E8, 1:100) for 60 min at 37°C. The cells were washed three times with 0.05% Tween 20 in PBS (PBST), and incubated with the secondary antibody (HRP-labeled goat anti-mouse IgG, 1:2000, Sigma, USA) for 60 min at 37°C. The cells were visualized using the substrate 3-amino-9-ethylcarbazole (AEC).

### Immunofluorescence assay (IFA)

PK-15 cells infected with TGEV H strain (MOI of 0.1) were cultured for 36 h. The cells were fixed and blocked as described above and incubated with the primary antibody (mAb 5E8, 1:100) for 60 min at 37°C, after which they were washed three times with PBST. Subsequently, the cells were incubated with secondary antibody (fluorescein isothiocyanate [FITC]-labeled goat anti-mouse IgG, Kirkegaard & Perry, Gaithersburg, MD, USA). The nucleolus was visualized using a primary antibody (rabbit polyclonal antibody to nucleolin, 1:100, Abcam); the secondary antibody used was tetramethylrhodamine (TRITC)-labeled goat anti-rabbit IgG (1:200, Sigma). Nuclear staining with 4',6-diamidino-2-phenylindole (DAPI, Sigma) was performed as previously described [19]. The stained cells were washed three times with PBST and subsequently examined under a Leica TCS SP5 laser confocal microscope.

### Immunoprecipitation of N protein

Immunoprecipitation was conducted as previously described [17]. The lysate supernatant (500 μg) was incubated overnight at 4°C with 1 μg of mAb 5E8. Protein A/G PLUS-Agarose was added to this mixture according to the manufacturer's instructions. After washing four times with lysis buffer, the immunoprecipitated proteins were analyzed by western blotting using mAb 5E8. A lysate from mock-infected PK-15 cells was used as a control. Western blotting was performed as previously described [17].

### Polypeptide design and coupling

Seven peptides spanning aa 142–240, six peptides spanning aa 184–203, ten peptides spanning aa 241–382, and five peptides spanning aa 241–260 were synthesized by GL Biotech (Shangai, China) (Table 2). Approximately 4 mg of the peptides (aa 189–202 or aa 246–257) coupled with keyhole limpet hemocyanin (KLH) (aa 189-202-KLH or aa 246-257-KLH) or bovine serum albumin (BSA) (aa 189-202-BSA or aa 246-257-BSA) was synthesized by GL Biotech.

**Table 2:** Sequences for the synthesis of overlapping peptides based on the amino acid sequence of the TGEV N protein.

Residues	Amino acid sequence	Residues	Amino acid sequence
142–161	DGKVPGEFQLEVNQSRDNSR	255–274	SSANFGDSDLVANGSSAKHY
156–175	SRDNSRSRSQSRSRSRNRSQ	269–288	SSAKHYPQLAECVPSVSSIL
170–189	SRNRSQSRGRQQSNNKKDDS	283–302	SVSSILFGSYWTSKEDGDQI
184–203	NKKDDSVEQAVLAALKKLGV	297–316	EDGDQIEVTFTHKYHLPKDD
198–217	LKKLGVDTEKQQQRSRSKSK	311–330	HLPKDDPKTEQFLQQINAYA
212–231	SRSKSKERSNSKTRDTTPKN	325–344	QINAYARPSEVAKEQRKRKS
226–240	DTTPKNENKHTWKRT	339–358	QRKRKSRSKSAERSEQEVVP
184–198	NKKDDSVEQAVLAAL	353–372	EQEVVPDALIENYTDVFDDT
189–203	SVEQAVLAALKKLGV	367–382	DVFDDTQVEMIDEVTN
191–203	EQAVLAALKKLGV	241–255	AGKGDVTRFYGARSS
189–202	SVEQAVLAALKKLG	246–260	VTRFYGARSSSANFG
189–201	SVEQAVLAALKKL	246–257	VTRFYGARSSSA
190–202	VEQAVLAALKKLG	246–256	VTRFYGARSSS
241–260	AGKGDVTRFYGARSSSANFG	247–257	TRFYGARSSSA

### Indirect ELISA

Enzyme-linked immunosorbent assay (ELISA) plates were coated with purified recombinant protein (1 μg/well) or synthesized peptide (2 μg/well) at 4°C overnight and blocked with 5% skim milk at 37°C for 1 h. The plates were incubated with the culture supernatants of the 5E8 and 3D7 clones at 37°C for 1 h. HRP-labeled goat anti-mouse IgG (1:2000, Sigma) was used as the secondary antibody at 37°C for 1 h and the reaction was terminated with 2M H_2_SO_4_.

### Animal immunization with aa 189-202-KLH or aa 246-257-KLH peptides

Four 8-week-old specific pathogen free (SPF) BALB/c mice were subcutaneously (s.c.) immunized with aa 189-202-KLH or aa 246-257-KLH (50 μg per mouse) emulsified in complete Freund’s adjuvant (Sigma). The mice were boosted with aa 189-202-KLH or aa 246-257-KLH emulsified in incomplete Freund’s adjuvant (Sigma, USA) at two weeks after the first immunization. The mice were immunized four times. Serum samples from the mice were collected at 0, 14, 28, 42, and 56 days post-immunization. Antibody was detected using ELISA plates coated with aa189-202-BSA or aa246-257-BSA (2 μg/well).

### Immunohistochemistry (IHC) assay

The IHC assay was performed as previously described [20]. Slides were incubated with mAb 5E8 (1:100) at 4°C overnight and subsequently reacted with HRP-labeled goat anti-mouse IgG (1:2000, Sigma) for 1 h. Immunocomplexes were detected using the 3,3’-diaminobenzidine (DAB) liquid substrate system.

### 3D model of the TGEV N protein

Using PyMOL software, the spatial distribution of the identified epitope was analyzed on a 3D model of the TGEV N protein using the SWISS-MODEL server [21].

### Ethics approval

This study was approved by Harbin Veterinary Research Institute and was performed in accordance with animal ethics guidelines and approved protocols. The animal Ethics Committee approval number is Heilongjiang-SYXK-2006-032.

### Animal care and use

Animals used in the experiment were fed in individual ventilated cages (IVCs). Each cage housed four mice. The animals were fed sterilized food consisted of whole nutrient pellet feed, which had been sterilized by radiation. The water was high-temperature-sterilized water that had been filtered three times. The bottles of water were cleaned and disinfected before use and were replaced every day. Poplar sawdust sterilized by radiation was used as the pad (replaced every three days). We monitored the physical condition of the animals two times (8 a.m. and 16 p.m.) a day during our experiment. No animal was severely ill or died prior to the experimental endpoint. At end of the experiment, we euthanized all animals utilized in this study by CO2 inhalation; a secondary physical method of euthanasia (cervical dislocation) was used to ensure death.

If the animals had become ill, we would perform the following steps to minimize pain and distress: An ill mouse would first be isolated by placing in a new IVC with fresh sterilized pad, water and feed. The used IVC cage would be sterilized thoroughly. Then we would diagnose the disease of the ill mouse, which would be given proper treatment to minimize pain and distress. We have a protocol in place for early euthanasia/humane endpoints for animals. The protocol is: CO2 inhalation and then cervical dislocation. We would use the early euthanasia/humane endpoints if a mouse exhibits the following clinical signs: The mouse appears to be wasting and with severe pain and distress, including lack of movement, no consumption of food and water, with fur that lacks luster and is messy.

## Results

### Preparation of mAbs against the TGEV N protein

**Fig 1:**
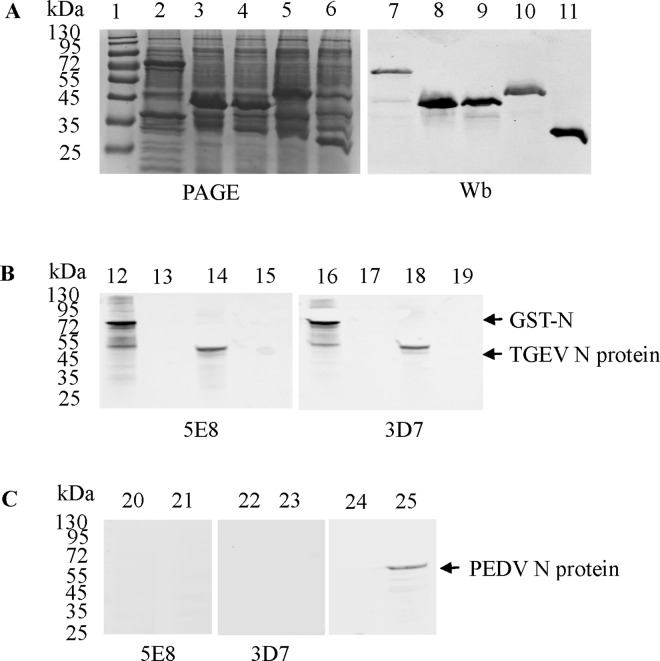
Preparation of mAbs against the N protein of TGEV. (A) Expression and purification of TGEV GST-N, GST-N1, GST-N2 and GST-N3 proteins. The proteins were visualized using PhastGel Blue R staining (lanes 1–6) or were detected after western blotting with a GST mAb (lanes 7–11). Lanes 2 and 7: GST-N protein. Lane 1: protein molecular weight marker. Lanes 3 and 8: GST-N1 protein. Lanes 4 and 9: GST-N2 protein. Lanes 5 and 10: GST-N3 protein. Lanes 6 and 11: GST protein. (B) Reactivity of the mAb 5E8 with the GST-N protein and the TGEV N protein. Lanes 12 and 16: GST-N protein. Lanes 13 and 17: GST protein. Lanes 14 and 18: cell lysates of TGEV-infected PK-15 cells. Lanes 15 and 19: cell lysates of mock-infected PK-15 cells. (C) Reactivity of 5E8 and 3D7 with PEDV N protein. Lanes 20, 22, and 24: cell lysates of PEDV-infected Vero E6 cells. Lanes 21, 23, and 25: cell lysates of mock-infected Vero E6 cells. The PEDV mAb was maintained in the lab.

The recombinant GST-N protein was expressed in pGEX-TGEV-N-transformed cells. The purified GST-N protein was recognized by the anti-GST mAb in western blotting (Fig 1). Three truncated N proteins (N1, N2 and N3) were also expressed and recognized by the anti-GST mAb using western blotting (Fig 1A). Two mAbs (5E8 and 3D7) against the TGEV N protein were produced using purified GST-N protein. The subtypes of mAb 5E8 and 3D7 were IgG2a and IgG2b, respectively. The mAbs 5E8 and 3D7 specifically recognized the recombinant GST-N protein and native N protein in TGEV-infected PK-15 cells in a western blotting assay but not in GST-treated and mock-infected PK-15 cells (Fig 1B). To further validate whether 5E8 and 3D7 react with PEDV, western blotting was used. As shown in Fig 1C, 5E8 and 3D7 did not react with the PEDV N protein.

### Identification the epitopes of mAbs 5E8 and 3D7

**Fig 2:**
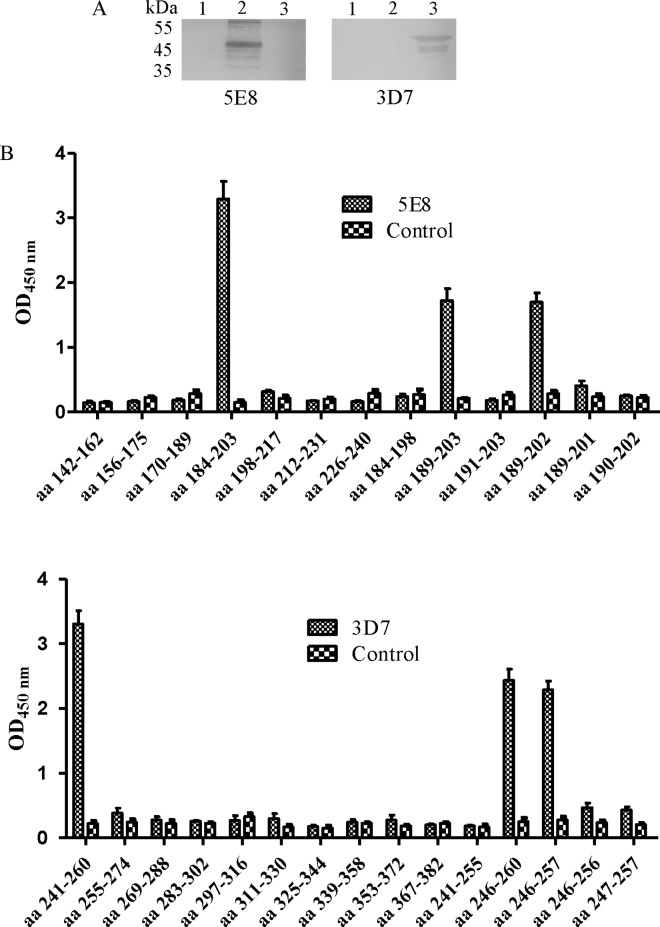
Identification of the epitopes of mAb 5E8. (A) Western blotting analysis of GST-N1, GST-N2 and GST-N3 proteins using mAb 5E8. Lane 1: GST-N1 protein; Lane 2: GST-N2 protein; Lane 3: GST-N3 protein. (B) Seventeen peptides were reacted with mAb 5E8 and fifteen peptides with 3D7 by peptide ELISA.

To identify the linear epitopes of 5E8 and 3D7, three truncated N proteins (N1, N2 and N3) were expressed respectively. Western blotting analysis revealed that 5E8 was reactive with GST-N2 (Fig 2A). Subsequently, seven overlapping polypeptides covering N2 were synthesized (Table 2). The results of the peptide ELISA demonstrated that 5E8 reacted with aa184-203 (Fig 2B). To further define the epitope of mAb 5E8, six overlapping polypeptides were subsequently synthesized (Table 2). Further indirect epitope ELISA experiments revealed that ^189^SVEQAVLAALKKLG^202^ was the core amino acid sequence of the 5E8 epitope (Fig 2B).

Western blot analysis showed that 3D7 was reactive with GST-N3 (Fig 2A). Subsequently, ten overlapping polypeptides covering GST-N3 were synthesized, and ELISA demonstrated that 3D7 reacted with aa 241–260 (Fig 2B). To define the epitope more precisely, five overlapping polypeptides were subsequently synthesized. Further indirect epitope ELISA experiments revealed that ^246^VTRFYGARSSSA^257^ was the core amino acid sequence of the 3D7 epitope (Fig 2B).

### Confirmation of epitopes in transfected PK-15 cells

**Fig 3:**
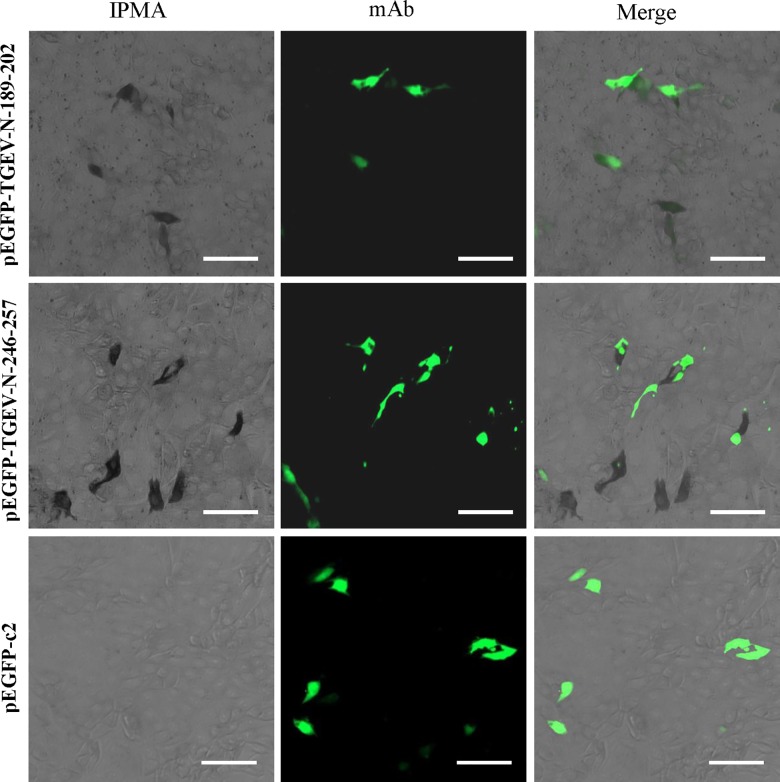
IPMA analysis of pEGFP-TGEV-N-189-202 reactivity with mAb 5E8 and pEGFP-TGEV-N-246-257 with mAb 3D7. Bar, 50 μm.

To confirm the epitopes of 5E8 and 3D7, PK-15 cells were transfected with pEGFP-TGEV-N-189-202 and pEGFP-TGEV-N-246-257. We observed that pEGFP-TGEV-N-189-202 reacted with mAb 5E8 and that pEGFP-TGEV-N-246-257 reacted with mAb 3D7 (Fig 3), indicating that the epitope recognized by mAb 5E8 was ^189^SVEQAVLAALKKLG^202^ and that the epitope recognized by mAb 3D7 was ^246^VTRFYGARSSSA^257^.

### Model of the TGEV N protein

**Fig 4:**
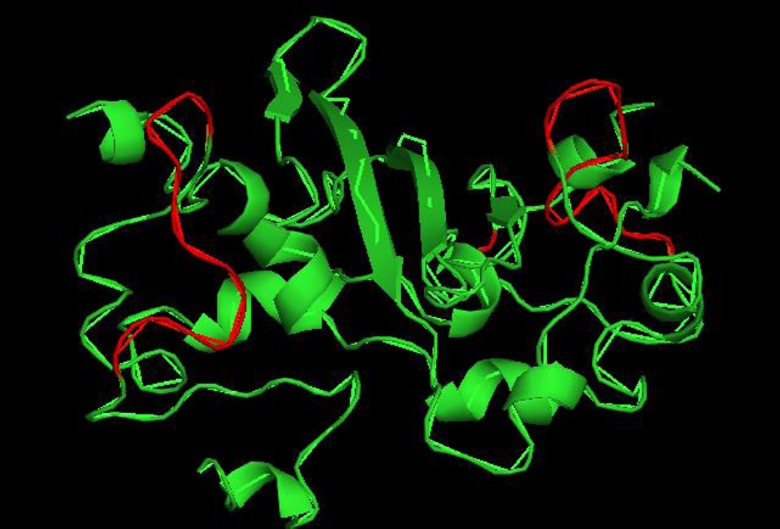
Location of an identified epitope in a predicted structure of the TGEV N protein CTD. The location of the epitope (aa 246–257, shown in red) for mAb 3D7 in the TGEV N protein CTD is highlighted.

The 3D structure of the TGEV N protein LKR was not available. However, the sequence of the TGEV N protein CTD (aa224-337) was searched against the SWISS-MODEL template library. The solution structure of the CTD of the SARS CoV N protein (SMTL id 2jw8.1.B) [22] was selected for model construction. The TGEV N protein was predicted to form a dimer via the CTD (Fig 4). The identified epitope recognized by mAb 3D7 (^246^VTRFYGARSSSA^257^) is located at the surface of the 3D structure of the TGEV N protein.

### Distribution of the N protein in PK-15 cells

**Fig 5:**
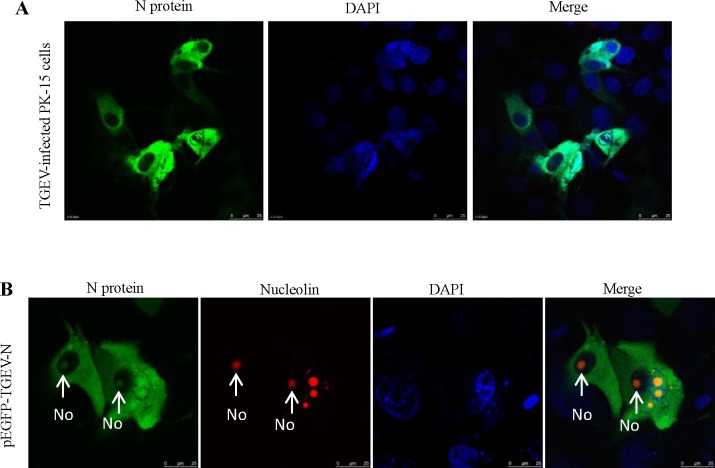
Subcellular localization of the N protein of TGEV. (A) IFA assay using mAb 5E8 in TGEV-infected PK-15 cells. (B) Localization of the TGEV N protein using pEGFP-TGEV-N in transfected PK-15 cells. The nucleolus (No) is indicated with an arrow (red).

The epitope ^189^SVEQAVLAALKKLG^202^ is well conserved among Alpha-CoVs, as shown in the sequence alignment in Table 3. Thus, 5E8 was used in the following experiments. IFA was used to verify the reactivity of mAb 5E8 with the N protein in TGEV-infected PK-15 cells. As shown in Fig 5A, mAb 5E8 exhibited reactivity with the N protein in TGEV-infected PK-15 cells and revealed that the N protein was distributed in the cytoplasm of PK-15 cells. To further analyze the subcellular localization of the N protein, pEGFP-TGEV-N (encoding GFP-N) was transfected into PK-15 cells. As shown in Fig 5B, the GFP-N protein was found in both the nucleolus and cytoplasm of PK-15 cells.

**Table 3:** Alignment of the sequences surrounding the identified epitopes of alpha-CoVs.

Viruses (GenBank accession number)	^189^SVEQAVLAALKKLG^202^	^246^VTRFYGARSSSA^257^
TGEV WH-1(ADY39745)	••••••••••••••	••••••••••••
TGEV virulent Purdue (ABG89328)	••••••••••••••	••••••••••••
TGEV TS (ABC72419)	••••••••••••••	••••••••••••
TGEV TFI (CAA84811)	••••••••••••••	••••••••••••
TGEV T014 (AAG30228)	••••••••••••••	••••••••••••
TGEV SC-Y (ABD97840)	••••••••••••••	••••••••••••
TGEV Purdue P115 (ABG89327)	••••••••••••••	••••••••••••
TGEV Miller M60 (ABG89310)	••••••••••••••	••••••••••••
TGEV Miller M6 (ABG89293)	••••••••••••••	••••••••••••
TGEV HYM-09-2 (ADC53234)	••••••••••••••	••••••••••••
TGEV FS772 (P05991)	••••••••••••••	••••••••••••
TGEV attenuate H (ABU49664)	••••••••••••••	••••••••••••
TGEV 96–1933 (AAC96008)	••••••••••••••	••K••S•••I••
Raccoon dog coronavirus GZ43 (ABO88143)	••••••••••••••	••K••••••I••
PRCV RM4 (CAA80841)	••••••••••••••	••••••••••••
PRCV ISU-1 (ABG89315)	••••••••••••••	••••••••••••
PRCV OH7269 (AKV62760)	••••••••••••••	•••••T•••G••
Chinese ferret badger coronavirus DM95 (ABO88144)	••••••••••••••	••K•••••••••
CCoV NTU336 (ADB28913)	••••••••••••••	••K•••••••••
CCoV K378 (CAA47246)	••••••••••••••	••K•••••••••
CCoV 430 (ACJ64181)	••••••••••••••	••K•••••••••
CCoV 1–71 (BAC65328)	••••••••••••••	••K•••••••••

Dots indicate identical residues.

### Immunoprecipitation of the TGEV N protein

**Fig 6:**
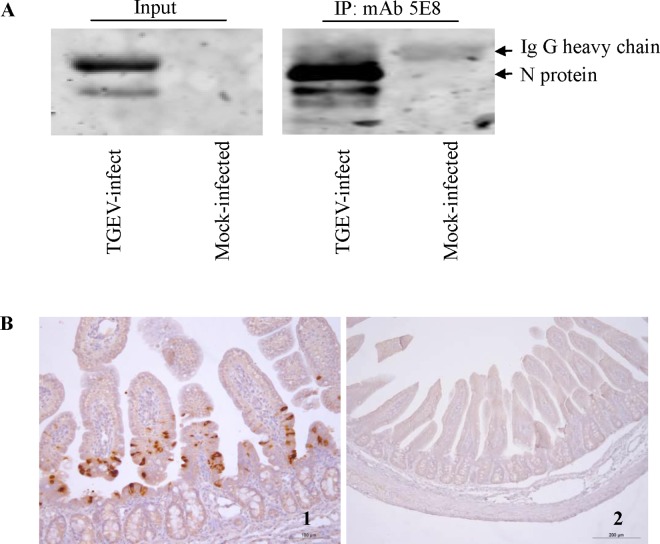
Application of the generated mAb 5E8 in various immunological assays and techniques. (A) Immunoprecipitation and western blotting analysis of the N protein in TGEV-infected PK-15 cells. (B) IHC assay using the mAb 5E8 in the small intestines of TGEV-inoculated animals. 1: TGEV-inoculated small intestine; 2: mock-inoculated small intestine.

An immunoprecipitation assay was employed to elucidate whether the TGEV N protein could be precipitated from TGEV-infected PK-15 cells using the mAb 5E8. MAb 5E8 precipitated the TGEV N protein from TGEV-infected PK-15 cells but not from mock-infected PK-15 cells (Fig 6A).

### The mAb 5E8 recognizes the N protein *in vivo*

IHC assay was utilized to ascertain whether mAb 5E8 could recognize the N protein in the small intestines of animals inoculated with TGEV. As shown in Fig 6B, the TGEV N protein was recognized by mAb 5E8 in the small intestines of animals inoculated with TGEV but not in control animals. In the figure, the primary region of staining is located in the goblet cells of villi in the small intestine.

### Antibody responses to epitopes

**Fig 7:**
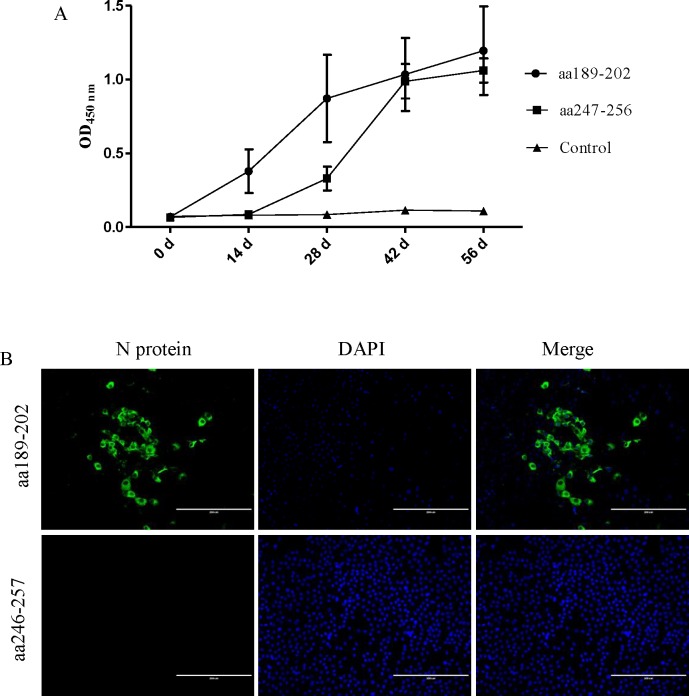
Antibody responses to aa 189–202 and aa 246–257 epitopes. (A) Humoral responses elicited by aa 189–202 and aa 246–257 epitopes. (B) Antibody responses elicited by aa 189–202 and aa 246–257 epitopes to determine their reaction with the TGEV virus in PK-15 cells.

BALB/c mice were inoculated with aa 189-202-KLH or aa 247-256-KLH at 2-week intervals. Serum samples were collected at 0, 14, 28, 42, and 56 days after the first immunization. Indirect peptide ELISA was used to examine the humoral responses elicited by the aa 189–202 and aa 247–256 epitopes. On day 14, the immunized group showed a detectable level of antibody, whereas sera collected from the control group inoculated with PBS did not show any significant level of immune response (Fig 7A). Increases in immune antibodies were observed in the sera of the groups immunized with aa 189-202-KLH or aa 246-257-KLH, collected on day 28 (14 days after the first boost). On day 56, the group immunized with aa 189-202-KLH or aa 246-257-KLH showed the highest antibody levels. Furthermore, IFA results showed that the antibody against aa 189–202 reacted with the N protein of TGEV-infected PK-15 cells. In contrast, the antibody against aa 246–257 did not react with the N protein of TGEV-infected PK-15 cells (Fig 7B).

### Establishment of the IPMA method for detection of the TGEV pathogen

**Fig 8:**
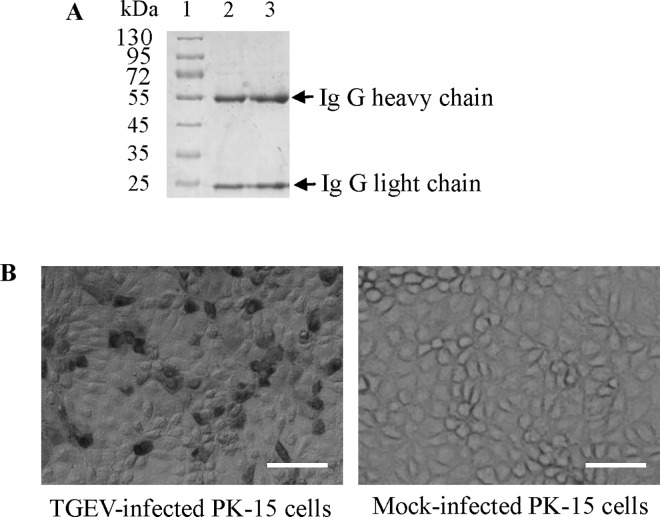
Optimization of the IPMA method for TGEV detection. (A) Purification of mAb 5E8 IgG, Lane 1: protein marker, Lanes 2 and 3: purified IgG. (B) IPMA method for TGEV detection in PK-15 cells using purified IgG. Bar, 50 μm.

The IgG of mAb 5E8 was purified using HiTrap^TM^ protein G HP (Fig 8A). The IPMA method was optimized for TGEV detection. The TGEV-infected PK-15 cells (10^3^ TCID_50_, 36 h post-infection) were fixed with paraformaldehyde (4%) for 20 min at 4°C. Subsequently, the cells were blocked with 5% skim milk at 37°C for 1 h. The optimum concentration of the primary antibody (purified 5E8 IgG) was 0.5 ng/μL, and the dilution of the secondary antibody was 1:2000. IPMA revealed red-brown staining in TGEV-infected PK-15 cells (Fig 8B).

## Discussion

Although TGEV can be lethal to piglets, it is an excellent model for studying CoV biology [23–25]. Mapping the epitopes of CoV viral proteins could enhance our current understanding of the antigenic structure and function of this virus. Although the N protein of TGEV has been instrumental in the diagnosis of TGEV [26–28], its biology remains unknown. The N protein of TGEV plays an important role during the life cycle of the virus and also undergoes caspase-mediated proteolysis, suggesting that the N protein can be targeted for destruction by the host cell’s death machinery [29]. Furthermore, the N proteins of CoVs are conducive to template switching and are required for efficient transcription [9]. TGEV N epitopes also elicit T helper cells that collaborate during *in vitro* antibody synthesis [30]. To understand the multiple functions of the TGEV N protein and to elucidate the mechanism involved in TGEV replication, mAbs against this protein are needed. The availability of specific antibodies against the TGEV N protein might also facilitate further studies on viral biosynthesis.

In the present study, we obtained 5E8 and 3D7, two mAbs against the TGEV N protein, using a recombinant GST-N protein. Combining experiments with truncated N proteins (N1, N2 and N3) and peptide scanning, two epitopes recognized by mAbs 5E8 and 3D7 corresponding to aa 189–202 (SVEQAVLAALKKLG) and aa 246–257 (VTRFYGARSSSA) in the TGEV N protein were subsequently identified. In experiments confirming these epitopes, IPMA and GFP did not merge very well (Fig 3), potentially reflecting the factthe dark brown staining of IPMA blocks the green fluorescence of IFA in some cells. Thus, the green fluorescence was not obvious. In the peptide ELISA analysis, mAb 5E8 did not react with aa 189–201 and aa 190–202 (Fig 2), indicating that S189 and G202 are key residues for the activity of the aa189-202 epitope. In addition, mAb 3D7 did not react with aa 245–257 and aa 246–256 in the peptide ELISA analysis (Fig 2), indicating that V246 and A257 are key residues for the activity of epitope of aa 246–257. The previously identified three linear epitopes on the TGEV N protein, namely, aa 46–60, aa 272–286, aa 321–335 [30], do not overlap with the epitopes recognized by mAbs 5E8 and 3D7. The results of western blotting demonstrated that mAbs 5E8 and 3D7 could recognize the native TGEV N protein.

The TGEV N protein has four distinct domains based on amino acid sequence comparisons: IDRs (aa 1–28; aa 334–382), an NTD (aa 29–156), an LKR (aa 157–223), and a CTD (aa224-337) [31, 32]. The LKR, an intrinsically disordered middle region separating the NTD and the CTD, is also known as the SR-rich domain because of the abundance of serine and arginine residues [33]. This domain is capable of direct interaction with viral RNA [34], and potential phosphorylation sites have been described in the LKR of CoV N proteins [35–37]. The phosphorylation site is believed to be a high-affinity region important for the binding of N to the nuclear ribosomal protein [38]. Previous studies have examined the function of the LKR in CoV N protein oligomerization, and mutational analyses have shown that an LKR motif within aa 184–196 is crucial for N protein oligomerization [39]. The LKR might play an essential role in the transformation of CoV N proteins between dimer and multimer configurations [14]. We speculate that the TGEV N protein LKR acts in modulating viral transcription and translation. In the present study, the identified linear epitope of mAb 5E8 is located in the LKR of the TGEV N protein. To our knowledge, mAb 5E8 is the first antibody to recognize an epitope in the LKR region, and it could be used in the future to evaluate the functions of this domain in viral transcription and translation.

Previous studies have revealed that the NTD and CTD of CoV N proteins are responsible for RNA binding and oligomerization, including SARS-CoV [40, 41], HCoV-229E [42], and avian infectious bronchitis virus (IBV) [42–46]. The structures of the NTD and CTD have been investigated [46–48]. As the CTDs of the CoV N proteins mediate self-association in oligomers formation, the CTD is a good target for mutagenesis research on disrupting CoV N protein self-association and virion assembly [15, 16]. In the present study, the identified linear epitope of mAb 3D7 was found to be located in the CTD of the TGEV N protein, and this mAb could be used for elucidating the function of this domain.

Sequence analysis showed that the identified epitope aa 189–202 (mAb 5E8) in the N protein is highly conserved, with 100% identity, among alpha CoVs, (Table 3). The high conservation of the aa 189–202 epitope in the N protein is advantageous to the development of mAb 5E8 for diagnostic technologies. Indeed, the mAb 5E8 generated against the TGEV N protein could be applied to various assays. For example, mAb 5E8 was used to reveal the location of the N protein during TGEV replication and to immunoprecipitate the N protein from lysates of TGEV-infected PK-15 cells (Fig 6A). The capacity of mAb 5E8 to immunoprecipitate the N protein will promote further studies on interactions of the N protein with viral and cellular proteins. Furthermore, because mAb 5E8 is effective in IHC assays, this mAb will benefit studies on the distribution of TGEV in infected animals and the pathogenesis of the virus *in vivo*. Interestingly, in the present study we observed TGEV N proteins of different sizes in TGEV-infected PK-15 cells (Fig 6A), suggesting that the N protein was cleaved by host or viral proteases during viral replication.

In virus-infected cells, CoV N proteins are present in the cytoplasm alone or in both the cytoplasm and the nucleolus [13, 49, 50]. In the present study, using the recombinant expression vector pEGFP-TGEV-N, the distribution of the N protein was investigated. We demonstrated the N protein was localized in the cytoplasm and nucleolus of transfected PK-15 cells (Fig 5). In addition, we detected the distribution of the TGEV N protein using mAb 5E8 in TGEV-infected PK-15 cells. However, no nucleolar localization of the N protein was observed using mAb 5E8 in TGEV-infected cells. This finding reflects the higher concentration of the antigen in the nucleus, which might block recognition [51]. Previous studies have reported that N protein nucleolar localization could not be detected in SARS-CoV-infected cells using antibodies against the N protein [52]. The mechanism of TGEV N protein localization to the nucleolus was not determined in the present study. Active transportation is required for proteins or nucleic acids greater than 40 kDa across the nuclear pore [53]. During this process, macromolecular transport across the pore not only requires energy but also a nuclear localization signal (NLS) [54, 55]. The TGEV N protein is predicted to possess two NLSs: ^340^RKRK^343^ (pat 4) and ^199^KKLGVDTEKQQQRSRSK^215^ (bipartite). Therefore, the TGEV N protein might be transported into the nucleus via an active pathway using an NLS.

## Conclusions

In summary, two specific mAbs against the TGEV N protein, 5E8 and 3D7, were produced in the present study, and two linear B-cell epitopes, located in the N protein LKR and CTD, were successfully identified. Subcellular localization of the N protein was observed using mAb 5E8, which was also used to immunoprecipitate the N protein from lysates of TGEV-infected PK-15 cells. The mAb 5E8 is a useful tool for investigating the antigenic properties of the N protein and developing a diagnostic test for TGEV infection. Furthermore, these antibodies are relevant to the further understanding of the replication mechanism of TGEV.
